# Can action tendencies be counteracted by inducing incompatible emotions? Considering instances of anxiety and anger

**DOI:** 10.1002/brb3.3247

**Published:** 2023-09-07

**Authors:** Emma Elkjær, Peter Kuppens, Mai B. Mikkelsen, Mia S. O'Toole

**Affiliations:** ^1^ Department for Psychology and Behavioral Sciences Aarhus University Aarhus Denmark; ^2^ Faculty of Psychology and Educational Sciences KU Leuven Leuven Belgium

**Keywords:** action tendencies, appraisals, emotion experience, emotion induction, incompatible response hypothesis

## Abstract

**Background:**

The *incompatible response hypothesis* suggests that emotions and other affective states can counteract each other when incompatible. With the present study, we focused on two negative emotions (anger and anxiety) associated with different action tendencies (approach vs. avoidance). Specifically, we wanted to investigate if an anxiety manipulation, subsequent to an anger manipulation, would show a counteracting effect of the approach action tendencies associated with the initial anger manipulation and vice versa for anxiety and avoidance tendencies.

**Methods:**

We conducted a preregistered online experiment (*N* = 173). We evaluated changes from when the individual (1) was presented with a task in relation to a specific goal (e.g., anxiety induction: recordings of students’ view on climate changes), (2) received a subsequent emotion induction framed within an unrelated task and goal (e.g., anger induction: student feedback on changes to the economic student compensation system), (3) after which they were asked to return to the initial task (e.g., from the anger induction back to the anxiety induction). Primary outcomes included visual and verbal measures of action tendencies, and secondary outcomes included appraisals and emotion experience.

**Results:**

The results showed no evidence of a counteractive effect by inducing emotions unrelated to the initial task and with incompatible action tendencies. Rather, results pointed to spill‐over effects, which should be seen in light of the anger conditions resulting not only in increase anger and irritability but also anxiety and nervousness.

**Conclusions:**

The lack of counteractive effects could be due to either the mixed emotions induced by the anger condition or the compatibility of motivational context (i.e., threat) of anxiety and anger. Future research needs to refine the *incompatible response hypothesis*, honing the ways in which incompatibility is needed for emotion alteration, for instance by investigating the role of the motivational context.

## Graphical abstract

Research is lacking on how emotional states need to be incompatible for counteraction to take place. The results of this study showed no evidence of a counteractive effect by inducing emotions unrelated to an initial task and with incompatible action tendencies. The lack of counteractive effects could be due to the compatibility of motivational context (i.e., threat) of anxiety and anger.

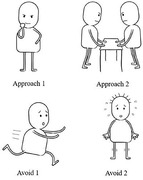

Emotions involve appraisals or meaning condensation of the personal relevance of a situation in relation to the individual's goals (Barrett, 2014; Moors, 2014). They are believed to be functional by motivating the individual to take actions toward obtaining current goals, evidenced as action tendencies (Scherer & Moors, 2019). Action tendencies are typically separated into approach (i.e., moving toward) and avoidance (i.e., moving away) impetuses (Frijda et al., 1989). Within a given culture, distinct emotional states are often associated with specific action tendencies. In Western societies, for instance, the experience of anger is often associated with a perceived provocation, resulting in approach behavior such as aggression (Lutz & Krahé, 2018). Anxiety, on the other hand, often reflects perceived danger, resulting in avoidance behavior such as fleeing or freezing (Frijda et al., 1989; Lutz & Krahé, 2018). However, although some regularities can be found, it should be noted that there is no one‐to‐one relationship between emotions and action tendencies (Wendt et al., 2017).

Emotions are not always contextually appropriate, and even when congruent with short‐term goals, they may at times conflict with long‐term goals (Berrios et al., 2015; Moors et al., 2017). The ability to alter one's emotions is thus critical. One way of regulating one's emotions may be the activation of *different* emotions. Indeed, according to the *incompatible response hypothesis*, competing affective states may alter behavioral tendencies such as aggressive acts (Baron, 1976, 1984). Even though the incompatible response hypothesis has been known for years and many laypeople and researchers implicitly seem to start from an incompatible assumption (e.g., inducing happiness should counteract sadness), there is surprisingly little contemporary research on the topic. More importantly, investigations looking into potential underlying processes behind the effects of competing emotions are even more sparse. Recently it was suggested that emotions associated with opposing action tendencies (i.e., approach vs. avoidance) may counteract each other (Lutz & Krahé, 2018). Hence, it has been proposed that negative and positive emotions are not necessarily incompatible in the sense that they can both concern approach action tendencies (e.g., running toward something in happiness, fighting someone in anger; Krieglmeyer & Deutsch, 2013; O'Toole & Mikkelsen, 2021). Studies have shown that the induction of sadness (i.e., associated with avoidance tendencies) following an anger induction can counteract the initial anger response (i.e., associated with approach tendencies) (Lutz & Krahé, 2018; Zhan et al., 2015). This has been taken as support for the *incompatible response hypothesis*. However, neither Zhan et al. (2015) nor Lutz and Krahé (2018) measured approach or avoidance tendencies as such, rather they simply relied on self‐reported affect and aggressive behavior. In addition, sadness and anger may be said to be incompatible not only in terms of the type of action tendency they are associated with, but possibly also in terms of physiological changes (e.g., hyper or hypo‐activation; Etkin & Wager, 2007). Furthermore, whereas anger is often believed to concern the approach of a threat, sadness may be understood as withdrawal from reward seeking (Adams et al., 2006; Krieglmeyer & Deutsch, 2013; O'Toole & Mikkelsen, 2021). This actualizes the question of whether it suffices that action tendencies are incompatible in terms of type of motivational impetus (i.e., approach vs. avoidance) for a counteractive effect to appear. To test this, it would be relevant to look at the possible counteractive effect of fear or anxiety on anger and vice versa, since these emotions, although involving opposite action tendencies (i.e., avoidance vs. approach), could be said to involve shared physiology (Prather, 2018; Siegel et al., 2018), and, at least in some instances, the same motivational context (i.e., threat; O'Toole & Mikkelsen, 2021). In this regard, Zhan et al. (2015) found that fear did not counteract anger. However, only the counteractive effect of fear on anger was measured, and not the other way around. Although the study found that the induction of fear in fact increased the level of experienced anger, this was not evidenced and measured as motivational changes (e.g., action tendencies) or changes in other parts of the emotional process (e.g., appraisals and overall emotion experience).

Accordingly, as our primary objective, we wanted to investigate the extent to which the induction of anxiety subsequent to an anger induction *and* vice versa, would show counteractive effects evaluated as changes in action tendencies. As a secondary aim, we wanted to explore how the effect of such inductions may be associated with alterations not only in action tendencies but also in the individual's appraisals and the ways in which they experience their emotions (Kuppens et al., 2008; Lerner & Keltner, 2001; Scherer & Moors, 2019). Appraisal accounts of emotions often assume that current emotions influence how new situations are perceived such that the emotional response not only is elicited by an appraisal but also influences subsequent appraisals (Scherer & Moors, 2019). As such, a counteractive effect may be believed to happen as a result of an appraisal change. For instance, being afraid of spiders may result in an appraisal of a journey to Australia being unsafe. However, an incoming phone call in that moment from a good friend may co‐activate joy. This co‐activation of an emotion, even unrelated to the original situation, may change the individual's overall somatosensory sensations, which is believed to influence subsequent appraisals.

Furthermore, the induction of emotions may be hypothesized to change the emotional experience in several ways. For instance, it may change the overall number of experienced emotions. The number of experienced emotions is sometimes referred to as emotion co‐activation (EC), reflecting an emotional experience in which the individual reports the experience of multiple emotions at the same time. In terms of operationalization, EC has been determined by the number of emotions above a certain threshold (Grossmann et al., 2016; Grühn et al., 2013; O'Toole et al., 2020). The induction of emotions may also be experientially captured as a change in the dispersion of emotions, also known as emotion variability (EV). EV is often operationalized as the standard deviation from a mean calculated across several emotions at one or across more time points (Grühn et al., 2013). An increase in the number of emotions experienced simultaneously (i.e., co‐activation) has both been associated with lower distress (Kircanski et al., 2012) and increases in self‐harm behaviors (Andrewes et al., 2017). Mixed results also exist for EV, which is sometimes associated with better emotion regulation (Ebner‐Priemer et al., 2015) and sometimes with lower levels of well‐being (Houben et al., 2015). It remains to be investigated how the induction of an emotion, incompatible with an existing emotion, may manifest as changes in the person's emotion experience.

## 1 PRESENT STUDY

With the present study, we investigated two negative emotions thought to be associated with different action tendencies. Specifically, we tested if an anxiety manipulation, subsequent to an anger manipulation, would show a counteracting effect of approach action tendencies associated with the initial manipulation and vice versa. We did this by evaluating changes from when the individual (T1) was presented with a task in relation to a specific goal (e.g., anxiety induction), (T2) received a subsequent emotion induction framed within an unrelated task and goal (e.g., anger induction), (T3) after which they were asked to return to the initial task (e.g., from the anger induction back to the anxiety induction). According to a computer‐generated randomization list, participants were randomized to one of four conditions (anxiety–anger/anxiety‐control or anger–anxiety/anger‐control); (1) an anxiety induction followed by either (a) an anger induction or (b) a neutral induction or (2) an anger induction followed by either (a) an anxiety induction or (b) a neutral induction. Congruent with the *incompatible response hypothesis*, the primary hypotheses were as follows:

1. Participants in the anxiety–anger condition would show more attenuated avoidance action tendencies compared with the anxiety‐control condition, evidenced as a significant time (before vs. after the subsequent manipulation) × condition (anger vs. control condition subsequent to anxiety) interaction effect. Secondarily, we wanted to explore how the anxiety induction followed by the anger induction (i.e., anxiety–anger) resulted in changes in appraisals and emotion experience (i.e., EV and co‐activation).
2. Participants in the anger–anxiety condition would show more attenuated approach action tendencies compared with the anger‐control condition, evidenced as a significant time (before vs. after the subsequent manipulation) × condition (anxiety vs. control condition subsequent to anxiety) interaction effect. Secondarily, we wanted to explore if the anger induction followed by the anxiety induction (i.e., anger–anxiety) resulted in changes in appraisals and emotion experience (i.e., EV and co‐activation).

## 2 METHODS

The project was registered and approved by the Danish Data Protection Agency and the local IRB at Aarhus University and preregistered at *As Predicted* (18316). See https://aspredicted.org/blind.php?x=tw3mz8.

### 2.1 Participants

Participants were university students from various departments at a Danish University. Participants were above the age of 18 and proficient in Danish. Based on power calculations, using a mixed‐effects ANOVA, 2 (time; before vs. after the subsequent emotion induction) by 2 (conditions; manipulation vs. control), 90 participants within each context (anxiety vs. anger as the first emotion) were required to detect a small effect size (*d* = .3), with an alpha of .05 and a beta of .20 (G*power 3.0.10; Faul et al., 2007). We conservatively expected a small effect size and powered the study accordingly. Participants were recruited consecutively during the period from April to November 2019. The recruited sample consisted of 242 participants (81.1% women, mean age = 23.8, ranging from 19 to 45). A total of 113 participants were assigned to anxiety–anger and anxiety‐control conditions, however, due to 49 dropouts, the final number was 64, with 32 in each condition (79.7% women, mean age = 24.34, ranging from 20 to 45). A total of 129 participants were assigned to the anger–anxiety and anger‐control conditions; however, due to 20 dropouts, the final number was 109, with 49 in the active condition and 60 in the control condition (48.1% women, mean age = 23.5, ranging from 19 to 44). More participants in the anxiety–anger and anxiety‐control conditions dropped out compared with the anger–anxiety and anger‐control conditions (*p* < .001), possibly due to the immediate performance element in the two conditions where the anxiety induction came first (Kirschbaum et al., 1993). Due to the higher dropout rate in the anxiety–anger and anxiety‐control conditions, the final sample size was slightly smaller than planned. Participation was acknowledged by 20 DKK/3 USD donated to the Danish Cancer Association.

### 2.2 Procedures

We employed an overall cover story for the experiments, stating that the university sought input from students about different matters currently being discussed in the university council. These matters included initiatives regarding climate changes (used for the active anxiety condition), changes in the economic student compensation system (used for the active anger condition), and feedback on building materials for a new building (used for the control condition). The aim of the control condition was to present a task matched on time to the active conditions while not inducing the target emotions. At the end, we evaluated participants’ belief in the cover story. Participants were debriefed both in the survey and by phone. All cover stories were piloted on three students who were subsequently interviewed regarding their immediate emotional experience and their belief in the cover story.

#### 2.2.1 The anxiety–anger condition

(T1) Participants in the anxiety–anger condition were initially manipulated to be made anxious of an upcoming task (i.e., having to give an audio‐recorded talk on climate changes), (T2) after which half of the participants within this condition was subjected to a manipulation aimed at inducing anger (i.e., arguing against changes in the state economic student compensation system). As a control condition (i.e., anxiety‐control), the other half was asked to make a decision concerning a trivial matter (i.e., building‐material preference for a new building. (T3) Finally, participants returned to the original induced presented at T1 (i.e., climate talk), the aim being to investigate the effect of the T2 manipulation (i.e., inducing anger) on this same task.

#### 2.2.2 The anger–anxiety condition

The exact same manipulations were employed in this condition but in a different order. (T1) Participants in the anger–anxiety condition initially received information about proposed changes to Danish students’ state economic support system, aimed at inducing anger. They were given a task of writing down arguments against these changes, (T2) after which half of the participants within this condition was subjected to a manipulation aimed at inducing anxiety (i.e., task of giving an audio‐recorded talk on climate changes). The other half was subjected to the control condition. (T3) Finally, participants rated the originally induced task presented at T1 (i.e., economic changes), the aim being to investigate the effect of the T2 manipulation (i.e., inducing anxiety) on this same task. See the full script in Supporting Information section. The experiment lasted approximately 40 min.

### 2.3 Measures

#### 2.3.1 Outcome measures

**FIGURE 1:**
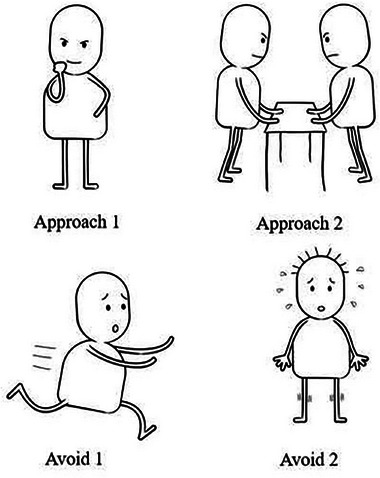
Depicted Action Tendencies (DAT) items.

Measures of *action tendencies* were the primary outcomes and the only outcomes for which a hypothesis was formulated a priori. Action tendencies were measured with (1) four pictures derived from the recently validated Depicted Action Tendency scale (O'Toole & Mikkelsen, 2021) depicting “avoid threat” and “approach threat” (see illustrations in Figure 1). Participants rated the extent to which they felt like the person in these pictures on a five‐point Likert scale; (2) four verbal items addressing approach (e.g., “How willing would you be to repeat the talk at an upcoming meeting at a university council?”) and avoidance tendencies (“How much do you wish you could avoid giving the talk?”) rated on a five‐point Likert scale. We evaluated this primary outcome by both visual and verbal means as prominent accounts of emotions (i.e., appraisal theories) propose that an individual's representation of action readiness precedes the verbal categorization in the emotion process (Scherer & Moors, 2019).

The remaining outcomes were secondary outcomes and subjected to exploratory analyses. *Appraisals* included motivational relevance: “How important is the matter of climate changes/keeping the economic system unchanged to you?” and “How important is your speaking performance to you?”. Motivational congruence: “How do you feel about having to give this talk? [anxiety]/What would it mean for you should the economic system change?” [anger]. Problem‐focused coping: “To what extent do you feel that there are things you can do to handle the situation?”. Future expectancy: “Do you think the speaking task/this issue will end well?” (Items were chosen and adapted from previous work; Fontaine et al., 2013; Verduyn et al., 2013). All items were rated on a 5‐point Likert scale except motivational congruence, which was rated from −2 (very bad) to +2 (very good). *Emotions* assessed included six negative emotions (i.e., anger, irritability, anxiety, nervousness, sadness, shame) and four positive emotions (i.e., happiness, interest, relaxation, content) rated on a 5‐point Likert scale. These emotions were chosen as they included the target emotions and are often used in studies aimed at capturing facets of emotion experience (see review by O'Toole et al., 2020). *Emotion experience* included; (1) negative EV calculated as the standard deviation over the person's mean of the negative emotions, where a larger standard deviation represents more variability (Grühn et al., 2013) and (2) negative EC calculated as the number of negative emotions rated above a threshold of 2 (on a scale from 1 to 5), where a greater number represents a higher degree of co‐activation (cf. Andrewes et al., 2017; Grühn et al., 2013).

### 2.4 Statistical analysis

As a manipulation check, to test if the conditions resulted in the expected emotional changes (i.e., elevated anger and anxiety in the two experiments), the anxiety and anger conditions at T1 were compared with independent samples *t*‐test. A successful anger manipulation was evidenced as a significant between‐group difference in terms of anger and irritability, favoring the anger condition, whereas a successful anxiety manipulation was evidenced as a between‐group difference in terms of anxiety and nervousness, favoring the anxiety condition. To explore the emotional effect of the subsequent manipulations on the target emotions, a 2 (time; T1 vs. T2) by 2 (condition; active vs. control) mixed‐effects ANOVA was conducted. To explore the association between outcome measures (i.e., action tendencies, appraisals, and emotion experience) correlation analyses were conducted to investigate the relationship between these indicators. Effect sizes were expressed as Pearson's *r*, with .1, .3, and .5, denoting a small, medium, and large effect size, respectively (Cohen, 1988).

Following the preregistered analytic strategy, the main hypotheses were tested with 2 (time; T1 vs. T3) by 2 (condition; active vs. control) mixed‐effects ANOVAs, determining the hypothesized larger attenuating effect of the active condition compared with the control condition. A mixed‐effects ANOVA was run for each of the outcomes. An attenuation of action tendencies in the anxiety setting was evidenced as a significant *time* × *condition* interaction effect on avoidance tendencies such that the anxiety–anger group would experience a larger attenuation of avoidance tendencies compared with the anxiety‐control group. In contrast, an attenuation of action tendencies in the anger setting was evidenced as a significant *time × condition* interaction effect on approach tendencies such that the anger–anxiety group would experience a larger attenuation of approach tendencies compared with the anger‐control group. Effect sizes were reported using partial eta squared and Cohen's *d* (Cohen, 1988; Richardson, 2011).

## 3 RESULTS

### 3.1 Associations between outcomes

Table 1 shows associations between measures of action tendencies, appraisals and emotion experience indicators. As evident from the results, DAT “approach threat” 1 and 2 were consistently and significantly associated with each other (*r* = .28/.54) and negatively associated with the verbal avoidance measure (*r* = −.16/−.19). DAT “approach threat 2” was also associated with the verbal approach item (*r* = .49/.45), the verbal avoidance item (*r* = −.25/−.42), and EC (*r* = .49/.44). In addition, the verbal approach measure was negatively associated with the verbal avoidance measure (*r* = −.43/−.76) and positively correlated with the appraisal of the importance of the topic (*r* = .34/.40) as well as EC (*r* = .42/.39). In terms of associations between DAT “avoid threat” 1 and 2, they were consistently associated with each other (large effects in T1/T3: *r* = .68/.75). DAT “avoid threat 1” was positively associated with EC (*r* = .27/.33). The verbal avoidance item was negatively correlated with the appraisal of the future expectancy (*r* = −.28/−.16).

**TABLE 1:** Correlations across all participants between action tendencies, appraisal, and emotion experience outcomes for T1 (below the diagonal line) and T3 (above the diagonal line).

			T3										
		M (SD)	DAT approach 1	DAT approach 2	DAT avoid 1	DAT avoid 2	V: approach	V: avoid	A: importance topic	A: coping	A: expectancy	Negative EV	Negative EC
T1	M (SD)	–	2.31 (1.32)	2.64 (1.42)	2.64 (1.42)	2.82 (1.41)	2.99 (1.59)	2.63 (1.31)	3.84 (1.07)	2.41 (1.03)	3.13 (1.07)	0.94 (0.48)	4.00 (1.81)
	DAT approach 1	2.36 (1.31)	–	.54^*^	−.02	.05	.20^**^	−.19^*^	.10	.03	.08	.09	.17^*^
	DAT approach 2	2.68 (1.46)	.28^*^	–	.04	.24^**^	.45^**^	−.42^**^	.20^**^	−.09	.08	.19^*^	.44^**^
	DAT avoid 1	2.62 (1.39)	−.07	.01	–	.75^**^	−.11	.14	.10	−.13	−.17^*^	.19^*^	.33^**^
	DAT avoid 2	2.91 (1.39)	−.06	.17^*^	.68^**^	–	.08	−.06	.20^**^	−.16^*^	−.20^*^	.22	.55^**^
	V: approach	3.08 (1.54)	.13	.49^**^	−.23^**^	.07	–	−.76^**^	.40^**^	.12	.16^*^	.21^**^	.39^**^
	V: avoid	2.91 (1.41)	−.16^*^	−.25^**^	.23^**^	−.02	−.43^**^	–	−.37^**^	−.13	−.16^*^	−.09	−.26^**^
	A: importance topic	3.91 (1.06)	.06	.14	.08	.−26^**^	.34^**^	−.17	–	.34^**^	.11	.08	.24^**^
	A: coping	2.55 (1.02)	−.03	−.37^**^	−.01	−.06	−.04	−.19	.14	–	.25^**^	−.23^*^	−.17^*^
	A: expectancy	3.35 (1.01)	.21^**^	.10	−.24^**^	−.15^*^	.21^**^	−.28^*^	.07	.19^*^	–	−.07	−.09
	Negative EV	1.04 (.45)	.00	.18^*^	.15	−.04	−.21^**^	.18	−.00	−.17^*^	−.02	–	.28^**^
	Negative EC	4.17 (1.74)	.02	.49^**^	.27^**^	−.15	.42^**^	.28^*^	.11	−.29^**^	−.06	.13	–

*Note*: V, verbal action tendency item; A, appraisal; coping, problem‐focused coping; expectancy, future expectancy.

Abbreviations: EC, emotion co‐activation; EV, emotion variability.

* =*p* < .05.

** =*p* < .01.

### 3.2 Manipulation check (T1)

The anxiety condition appeared to induce the target emotions and action tendencies, resulting in higher levels of anxiety and nervousness *within* this group. The anger condition resulted in more mixed emotions and action tendencies. *Between*‐group comparisons revealed that anger, *t*(170.4) = 16.23, *p* < .00, and irritability, *t*(107.13) = 11.01, *p* < .001, were both rated significantly higher at T1 in the anger condition compared with the anxiety condition. However, anxiety and nervousness were not rated higher at T1 in the anxiety condition. These emotional changes were reflected in the action tendencies as measured with the DAT, where both approach drawings were rated significantly higher in the anger than the anxiety condition (*p*s < .001), whereas this was not the case for the two avoidance drawings (*p*s > .05).

### 3.3 Emotional change from the first to the subsequent task (from T1 to T2)

#### 3.3.1 The anxiety–anger condition

The results showed a significant interaction effect of a large magnitude for both anger, *F*(1,62) = 41.03, *p* < .001, *η_p_^2^* = .40, *d* = 1.63, and irritability, *F*(1,62) = 56.96, *p* < .001, *η_p_^2^* = .48, *d* = 1.92, revealing a larger increase in both emotions for the anxiety–anger condition compared with the anxiety‐control condition. Concerning anxiety, *F*(1,62) = 1.06, *p* = .307, *η_p_^2^* = .02, *d* = .26, and nervousness, *F*(1,62) = 2.33, *p* = .132, *η_p_^2^* = .04, *d* = .39, no interaction effect was detected. Rather, a main effect of time for both nervousness, *F*(1,62) = 41.54, *p* < .001, *η_p_^2^* = .40, *d* = 1.64, and anxiety, *F*(1,62) = 9.55, *p* = .003, *η_p_^2^* = .13, *d* = .79, was revealed, showing that participants in both conditions became less nervous and anxious over time.

#### 3.3.2 The anger–anxiety condition

Both nervousness and anxiety were already present after the anger manipulation as the manipulation check showed. Significant interaction effects were revealed for both emotions nervousness, *F*(1,107) = 28.98, *p* < .001, *η_p_^2^* = .2,1, *d* = 1.04, and anxiety, *F*(1,107) = 21.81, *p* < .001, *η_p_^2^* = .17, *d* = .90. In both cases, a decline in nervousness and anxiety following the anxiety manipulation was evidenced, where the decline was larger for the control group.

**FIGURE 2:**
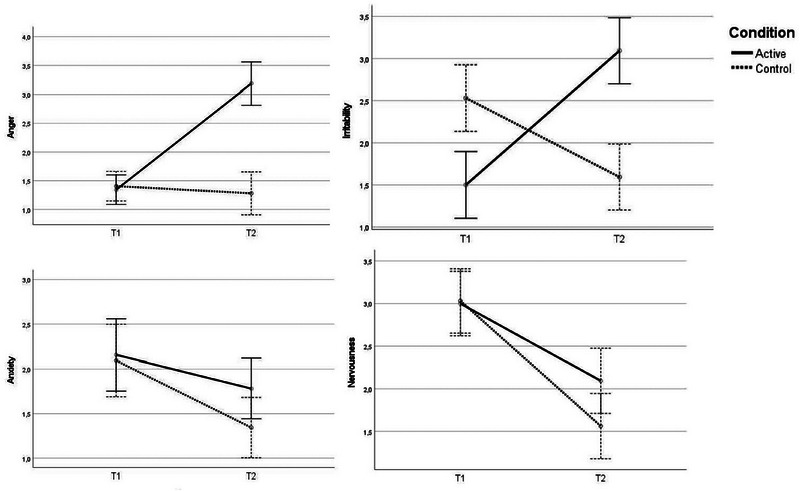
Emotional change from T1 to T2 in the anxiety‐first condition.

**FIGURE 3:**
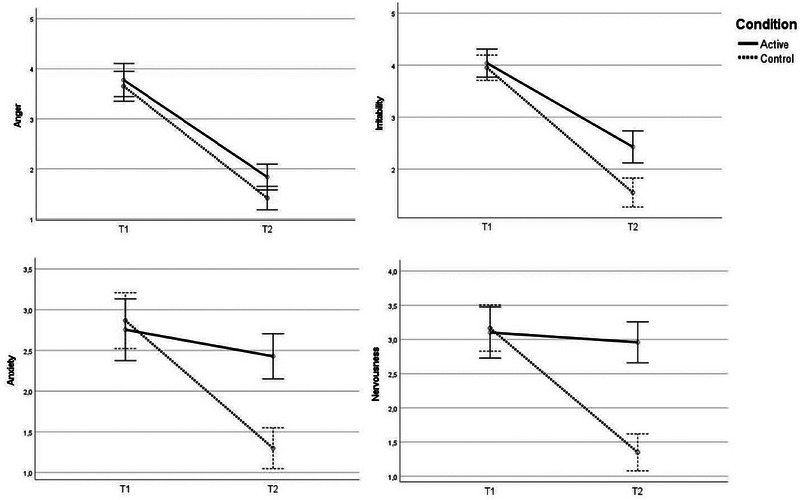
Emotional change from T1 to T2 in the anger‐first condition.

Results revealed a significant interaction effect for irritability, *F*(1,107) = 9.04, *p* = .003, *η_p_^2^* = .08, *d* = .58. Although both groups showed a decline in irritability following the anxiety manipulation, the decline was larger for the control group. No interaction effect was detected for anger, *F*(1,107) = 1.57, *p* = .213, *η_p_^2^* = .01, *d* = .24, rather, there was a main effect of time, *F*(1,107) = 314.86, *p* < .001, *η_p_^2^* = .75, *d* = 3.43, suggesting that participants in both conditions became less angry (see Table 2 and Figures 2 and 3).

**TABLE 2:** Manipulation checks of induced emotions and action tendencies at T1.

	*Anxiety first*	*Anger first*
	*N = 64*	*N = 109*
	*M (SD)*	*M (SD)*
Anger	1.38 (.72)	3.71 (1.17)
Irritability	2.02 (1.23)	3.99 (.95)
Anxiety	2.13 (1.13)	2.82 (1.33)
Nervousness	3.02 (1.06)	3.14 (1.32)
Approach 1	2.03 (1.14)	2.55 (1.37)
Approach 2	1.34 (.60)	3.45 (1.23)
Avoidance 1	2.78 (1.45)	2.52 (1.34)
Avoidance 2	2.67 (1.29)	3.05 (1.44)

#### 3.3.3 Results of the anxiety–anger condition

No significant interaction effects were detected on the DAT measures of “avoid threat” or the verbal measures of avoidance action tendencies. Thus, the anger manipulation did not exert an influence in terms of attenuating avoidance impetuses.

**FIGURE 4:**
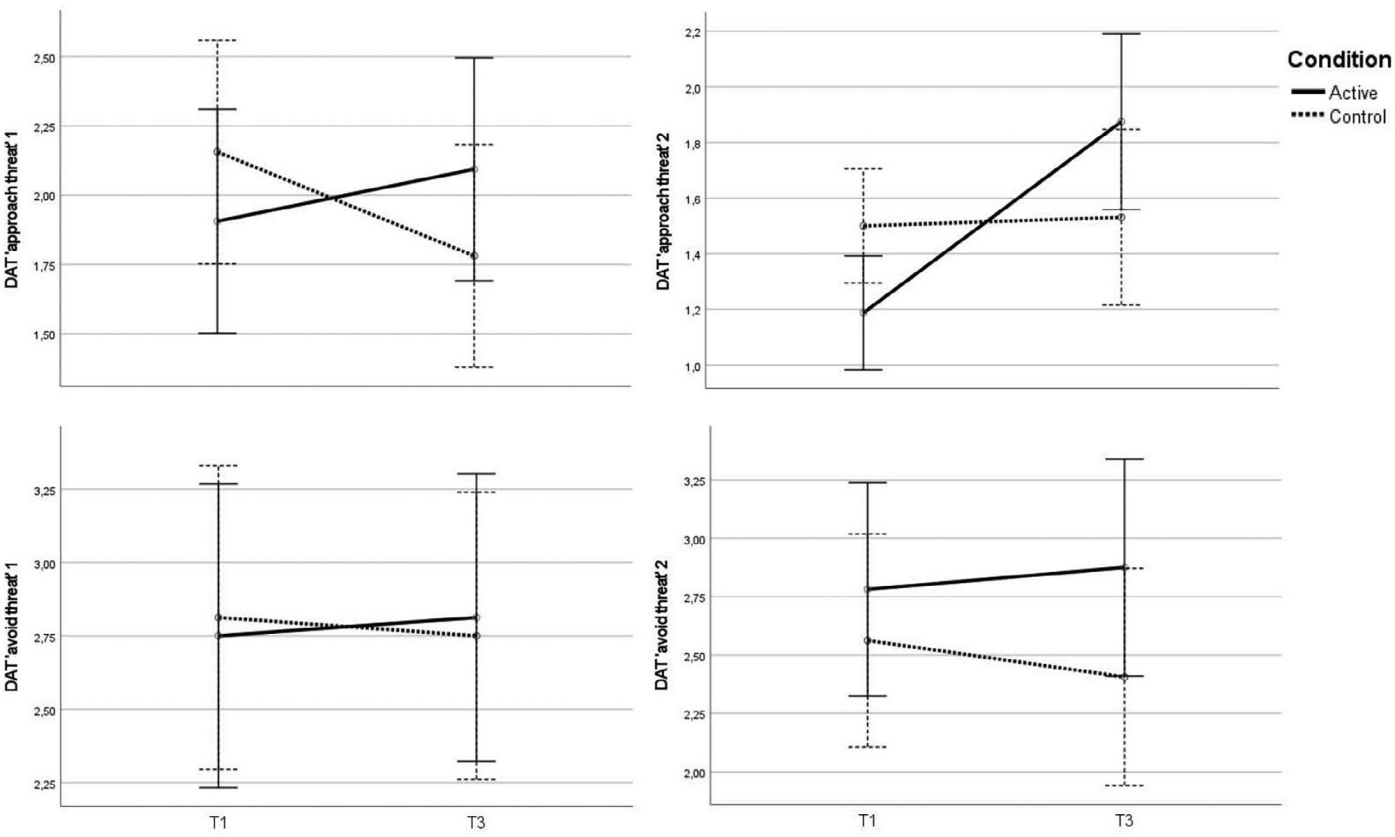
Changes in action tendencies from T1 to T3 in the anxiety‐first condition.

Concerning approach tendencies, a significant interaction effect of a medium magnitude, *F*(1,62) = 5.38, *p* = .024, *η_p_^2^* = .08, *d* = .59, was detected on the DAT “approach threat 1,” showing that approach impetuses increased the most in the anxiety–anger condition. Concerning the DAT “approach threat 2,” a significant interaction effect, corresponding to a large magnitude, was detected*, F*(1,62) = 11.92, *p* < .001, *η_p_^2^* = .16, *d* = .88, revealing a greater increase in approach tendencies for in the anxiety–anger condition compared with the anxiety‐control condition (see Figure 4).

The verbal measures of approach action tendencies did not differ over time between groups. None of the appraisal items or emotion experience indicators showed significant interaction effects (see Table 3).

**TABLE 3:** Means (M), standard deviations (SD), main, and interaction effects.

*Dependent variables*	Active group		Control group		Main effect (T1, T3)			Group (active vs. control) × time (T1 vs. T3)		
	T1	T3	T1	T3	*F*	*p*	*d*	*F*	*p*	*d*
**Results for the anxiety–anger (*N* = 32) anxiety‐control (*N* = 32) manipulations**										
DAT avoid 1 M (SD)	2.75 (1.48)	2.81 (1.36)	2.81 (1.45)	2.75 (1.41)	<.01	1.00	<.01	.15	.695	<.01
DAT avoid 2 M (SD)	2.78 (1.36)	2.87 (1.29)	2.56 (1.22)	2.41 (1.34)	.06	.813	<.01	.90	.347	.20
DAT approach 1 M (SD)	1.91 (1.09)	2.09 (1.12)	2.16 (1.20)	1.78 (1.2)	.60	.442	.20	**5.38**	**.024**	**.59**
DAT approach 2 M (SD)	1.19 (.39)	1.87 (.87)	1.50 (.72)	1.53 (.92)	**14.30**	**<.001**	**.97**	**11.92**	**<.001**	**.87**
V approach M (SD)	1.91 (1.06)	1.88 (1.1)	1.81 (1.15)	1.75 (1.11)	.52	.475	.20	.06	.811	<.01
V avoid M (SD)	3.59 (1.37)	3.34 (1.34)	3.53 (1.19)	3.56 (1.39)	.43	.516	.20	.71	.404	.20
A relevance topic M (SD)	3.75 (1.24)	3.76 (1.20)	3.72 (1.09)	3.63 (1.20)	.35	.555	.20	.35	.555	.20
A relevance task M (SD)	2.56 (1.16)	2.47 (1.16)	2.31 (1.15)	2.00 (1.20)	2.50	.119	.41	.72	.398	.20
A congruency M (SD)	2.72 (1.11)	2.50 (1.10)	2.41 (.88)	2.31 (1.00)	2.30	.135	.41	.37	.547	.20
A coping M (SD)	3.16 (1.08)	2.81 (1.06)	3.22 (.91)	2.59 (1.24)	**14.84**	**<.001**	**.97**	1.25	.268	.29
A expectancy M (SD)	3.09 (1.17)	2.88 (1.10)	3.06 (.98)	2.84 (1.05)	**5.43**	**.023**	**.59**	<.01	1.00	<.01
Negative EV M (SD)	.89 (.44)	.81 (.50)	.95 (.41)	.79 (.48)	3.66	.060	.20	.36	.553	.20
Negative EC M (SD)	2.53 (1.78)	2.81 (1.90)	3.22 (1.58)	3.00 (1.90)	.03	.858	<.01	2.07	.155	.35
**Results for the anger–anxiety (*N* = 49) + anger‐control (*N* = 60) manipulations**										
DAT avoid 1 M (SD)	2.45 (1.47)	2.67 (1.63)	2.58 (1.24)	2.45 (1.28)	.37	.546	<.01	**5.65**	**.019**	**.46**
DAT avoid 2 M (SD)	2.80 (1.49)	2.84 (1.48)	3.25 (1.39)	3.00 (1.43)	2.13	.147	.29	**4.13**	**.045**	**.41**
DAT approach 1 M (SD)	2.43 (1.37)	2.43 (1.32)	2.65 (1.38)	2.60 (1.43)	.08	.774	<.01	.08	.774	<.01
DAT approach 2 M (SD)	3.35 (1.35)	3.04 (1.47)	3.55 (1.12)	3.32 (1.30)	**9.64**	**.002**	**.59**	.18	.676	<.01
V Approach M (SD)	3.83 (1.31)	3.79 (1.35)	3.77 (1.29)	3.62 (1.46)	3.51	.064	.35	1.12	.292	.20
V Avoid M (SD)	4.17 (.91)	3.88 (.94)	3.90 (1.05)	3.85 (1.07)	**10.07**	**.002**	**.63**	**5.04**	**.027**	**.46**
A relevance topic M (SD)	4.06 (1.04)	3.85 (.97)	3.98 (.95)	4.00 (1.00)	2.21	.140	.29	3.05	.084	.353
A congruency M (SD)	1.51 (.85)	1.67 (1.05)	1.72 (.94)	1.67 (.93)	.50	.480	<.01	1.79	.184	.29
A coping M (SD)	2.10 (.78)	2.27 (.92)	2.25 (.9)	2.20 (.92)	.93	.338	.20	3.20	.077	.35
A expectancy M (SD)	3.65 (.91)	3.44 (1.05)	3.40 (.96)	3.17 (1.03)	**14.27**	**<.001**	**.74**	.05	.831	<.01
Negative EV M (SD)	1.13 (.48)	1.08 (.46)	1.08 (.41)	.98 (.45)	**4.38**	**.039**	**.41**	.36	.550	<.01
Negative EC M (SD)	4.80 (1.2)	4.59 (1.35)	4.59 (1.35)	4.64 (1.42)	**9.60**	**.002**	**.59**	.78	.378	.20

*Note*: T1, pre‐manipulation; T3, post manipulation; V, verbal Item; V Approach: “How willing would you be to attend a demonstration concerning this matter?”; V avoid: “How willing would you be to fight for the cause of keeping the economic situation as is?”; A, appraisal; congruency, motivational congruency; coping, problem‐focused coping; expectancy, future expectancy. Significant results (p<.05) are in bold.

Abbreviations: EC, emotion co‐activation; EV, emotion variability.

#### 3.3.4 Results of the anger–anxiety condition

Concerning approach tendencies, the DAT measures showed no interaction effects. However, regarding DAT “approach threat 2,” there was a main effect of time, *F*(1,107) = 9.64, *p* = .002, *η_p_^2^* = .08, *d* = .60, suggesting that both groups felt less impetus to approach the threat over time.

**FIGURE 5:**
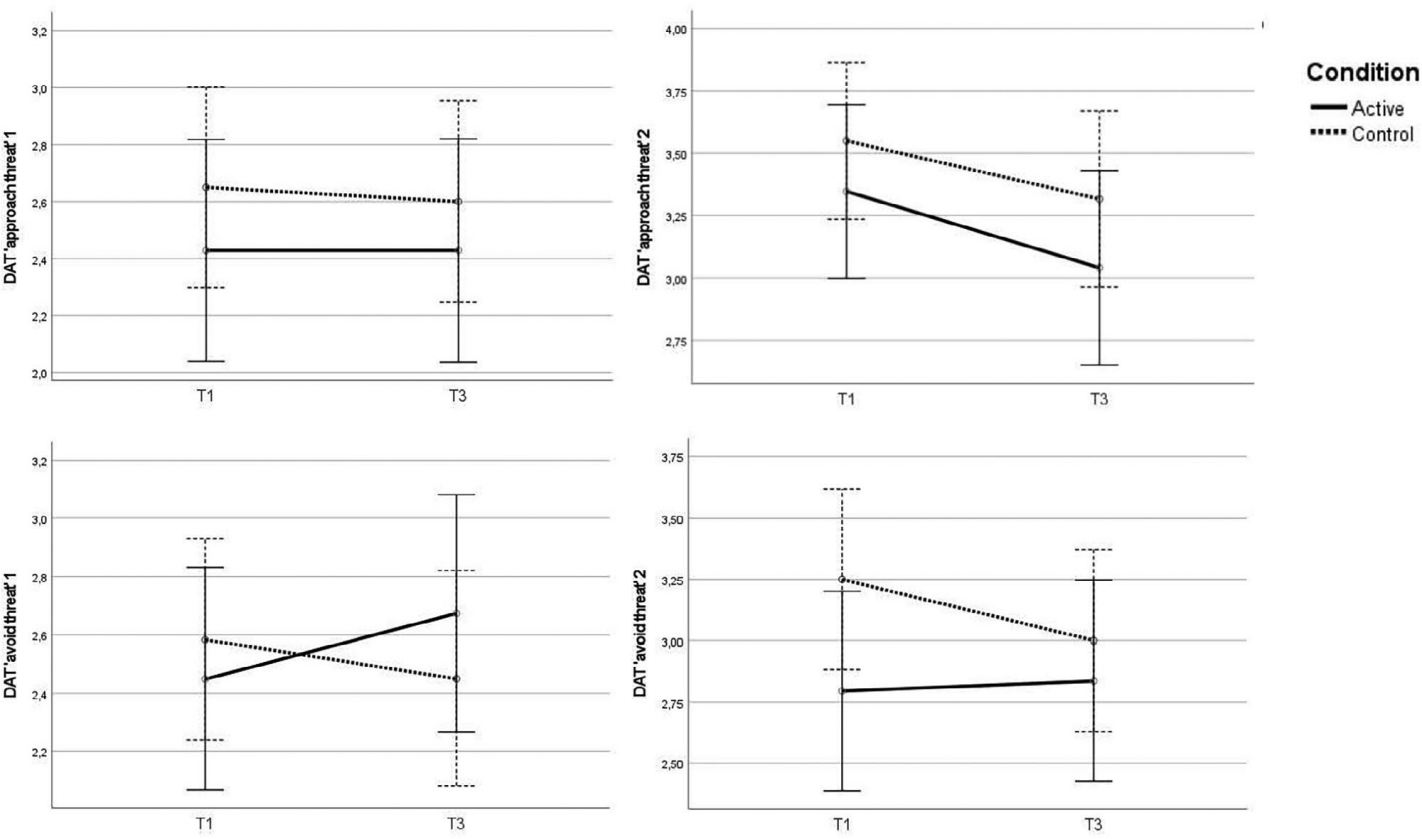
Changes in action tendencies from T1 to T3 in the anger‐first condition.

Concerning avoidance tendencies, significant interaction effects of small magnitudes were identified for both DAT “avoid threat 1,” *F*(1,107) = 5.65, *p* = .019, *η_p_^2^* = .05, *d* = .46, and “2,”, *F*(1,107) = 4.13, *p* = .045, *η_p_^2^* = .04, *d* = .39. Where the active condition increased in avoidance tendencies (i.e., DAT avoid 1 and 2), the control condition decreased (see Figure 5).

Regarding verbal measures of action tendencies, one significant interaction effect for the avoidance item of small magnitude was detected, *F*(1,107) = 5.04, *p* = .027, *η_p_^2^* = .05, *d* = .43. A larger decline in the approach item was detected for the active condition compared with the control condition. No other verbal measures of action tendencies were statistically significant. Neither emotion experience nor the appraisal items showed significant interaction effects (see Table 3).

Robustness of the results was checked by running the analyses with and without participants who believed in the cover story. The results did not change substantially by excluding participants (15.6% in the anxiety‐first conditions; 6.4% in the anger‐first conditions) who did not believe in the cover story.

## 4 DISCUSSION

This study aimed to explore the *incompatible response hypothesis* primarily in terms of action tendencies and secondarily in terms of appraisals, and emotion experiences. Although the initial emotion induction in anxiety–anger and anxiety‐control conditions resulted in the expected emotions (i.e., anxiety and nervousness), the anger–anxiety and anger‐control conditions appeared to increase not only anger and irritability but also anxiety and nervousness. Given that the subjective experience of anger is often intertwined with the experience of other negative emotions like fear or shame (Berkowitz, 1993), this is not a surprising finding. However, as we did not succeed in inducing distinct emotional states, our findings should be seen in light of these results, lacking emotional specificity. Specifically, our results cannot speak to the counteractive effect of anxiety on anger as distinct emotional states per se, but rather of this specific anxiety *induction* on the anger *induction* and vice versa.

Regarding action tendencies as evaluated visually (cf. O'Toole & Mikkelsen, 2021), the results did not reveal the expected counteractive effect in either of the active conditions compared to the control conditions (i.e., none of the expected *time* × *condition* interaction effects on action tendencies were statistically significant). Instead, an action tendency spill‐over effect was found. Specifically, rather than experiencing an attenuation of the action tendencies associated with the first manipulation (e.g., an attenuation of approach action tendencies in the anger–anxiety condition compared to the control), the second emotion induction (e.g., anxiety induction subsequent to the anger induction) resulted in *additional* action tendencies. Thus, an increase in action tendencies associated with the subsequent manipulation such that approach and avoidance action tendencies were experienced simultaneously.

There is a number of possible explanations for these findings. First, although contrary to our hypotheses, Lerner and Keltner (2001) argued for the effect of residual emotional changes. They state that emotion components, including cognition, physiology, and action, may linger and thereby affect the person's subsequent perception of a situation even when it is otherwise unrelated to the original situation that caused the emotion (so‐called appraisal tendencies). We note that these appraisal tendencies may also partly be responsible for the unexpected co‐activation we observed of anger and anxiety: Given the overlap in appraisal between both emotions, eliciting appraisals for the one emotion could also have induced the experience of the other emotion, leading to co‐activation. Second, our findings are congruent with a previous study showing that fear did not counteract anger (Zhan et al., 2015). These findings, however, could be said to go counter to different findings that sadness can counteract anger (Lutz & Krahé, 2018; Zhan et al., 2015). These seemingly contradictory findings may be attributable to sadness and anxiety pertaining to different motivational contexts. Indeed, sadness may arise within the context of *reward* (e.g., loss) and anxiety may arise within the context of *threat* (O'Toole & Mikkelsen, 2021). As such, inducing an emotion such as sadness could be hypothesized to be more likely to counteract anger since it likely involves the opposite motivational context. Moreover, the action tendencies typically associated with sadness and anxiety also differ. Where anxiety may motivate active avoidance (e.g., running away or escaping), sadness may be related to passivity and withdrawal. Even in cases where anxiety is associated with “freeze” reactions, this is considered a state of hyperarousal (Bracha, 2004; Roelofs, 2017; Roelofs et al., 2009). From this perspective, sadness may be hypothesized to hold stronger potential to counteract anger, as both anxiety and anger, unlike sadness, may mobilize the individual's arousal and motivation to take action. Albeit speculations, such interpretations of the present results and divergent findings in the literature, may point to the need for refining the *incompatible response hypothesis*, honing the ways in which incompatibility is needed for emotion alteration.

In addition to action tendencies, we conducted exploratory analyses evaluating changes in secondary outcomes including appraisals and emotion experience indicators. There were no significant interaction effects on any of the outcomes. One possibility may be that our verbal measures of appraisals did not capture participants’ actual appraisals or that the items were not sensitive to change. The same pattern was evidenced for verbal measures of action tendencies thus pointing to the visual material as potentially more sensitive to change and more directly mapping onto action tendencies.

Although we did not find the expected interactions, we investigated the correlations between the outcomes to test if these (i.e., action tendencies, appraisals, and emotion experience) reliably measured what we expected. Correlations among included measures can be found in Table 1, showing correlations in the expected direction, including positive correlations internally among measures pertaining to approach and likewise for measures pertaining to avoidance.

### 4.1 Limitations and future directions

First, the dropout rate was rather high in both groups, possibly resulting in completer data only for a biased portion of the sample. Reasons for dropout were not systematically obtained. If the dropout rate reflects intolerable anxiety levels, another less anxiety‐provoking speech topic could be chosen (e.g., a personal hobby). Second, slightly fewer participants than expected were included in the study, increasing the risk of a type‐II error. However, when looking at the nonsignificant interactions, the effect sizes were small (*η_p_^2^* ranging from .00 to .03), speaking against a considerable risk of a type‐II error. To ensure the robustness of the obtained results, well‐powered replication studies are needed. Third, not all participants were convinced by the cover story, but analyses performed without those participants who did not believe in the cover story showed no substantial changes in results. Fourth, the anger induction resulted in more emotions than anticipated (i.e., also anxiety and nervousness). Although analyses suggested that the subsequent anxiety induction was still effective, the spill‐over effect of motivations may reflect the spill‐over effect of emotions in those conditions. Given this finding in combination with the high dropout rate in the anxiety condition, other anxiety and anger manipulations may be considered in the future. Fifth, assessments of action tendencies, appraisals, and emotion experiences were conducted several times during the experiment potentially inducing demand effects. Sixth, the DAT measure and verbal items of approach and avoidance tendencies relied on self‐report measures. To address demand effects further, other measures of approach and avoidance tendencies, potentially less sensitive to such effects, such as the Manikin task (De Houwer et al., 2001) or a Joystick task (Rinck & Becker, 2007), could be considered. Seventh, considering that both the order of the manipulations and the setting (anxiety vs. anger) were between‐subject manipulations, the potential contribution of individual characteristics cannot be ruled out. Eight, given the number of statistical analyses run, it could have been relevant to correct for multiple comparisons. However, the study was not powered to detect a corrected *p‐*level and all significant effects exceeded a *d‐*value above .4, which is a considerable effects size for experiments of this type. At the same time, not correcting the *p‐*value increases the risk of a type‐I error, and it should be noted that the only result that would survive multiple comparison correction (e.g., *p* = .0125) would be the “DAT approach” outcome. Future research is clearly needed to ensure the robustness of the results.

## AUTHOR CONTRIBUTIONS

**Emma Elkjær**: Conceptualization, data curation; formal analysis; investigation; project administration; writing—original draft. **Peter Kuppens**: Conceptualization; methodology; supervision; writing—review and editing. **Mai B. Mikkelsen**: Conceptualization; formal analysis; investigation; supervision; writing—review and editing. **Mia S. O'Toole**: Conceptualization; data curation; formal analysis; methodology; supervision; writing—review and editing.

## CONFLICT OF INTEREST STATEMENT

The authors declare no conflict of interest.

## FUNDING INFORMATION

No funding was received.

### PEER REVIEW

The peer review history for this article is available at https://publons.com/publon/10.1002/brb3.3247.

## Supporting information

Supporting Information

## Data Availability

Data that support the findings of this study can be requested from the corresponding author.
