# Supplementary material for: Can action tendencies be counteracted by inducing incompatible emotions? Considering instances of anxiety and anger
Source: Brain Behav. 2023 Sep 7;13(11):e3247. doi: 10.1002/brb3.3247 (PMC10636385; doi:10.1002/brb3.3247)
Supplement: Supplementary file 1 — Supporting Information [file BRB3-13-e3247-s001.docx]

**Supplementary Material**

**Full manipulation procedure**

***Overall introduction***

“*Aarhus University highly values the involvement of students as much as possible in decision-making processes about current topics of discussion. This involvement of you as a student is in accordance with the vision of the university. For further information see* [link provided to the University’s newsletter].

*Your participation in this survey is therefore essential, as the decision-makers will carefully consider your answers. Today, Aarhus University wants your feedback on two subjects during this survey*” (note: two of three topics are mentioned depending on the condition):

- Anxiety condition: One topic includes university initiatives regarding climate changes for which the university explicitly seeks input from its students.
- Anger condition: Another topic concerns proposed national changes in the economic student compensation system for which the university would like student feedback.
- Control condition: Another topic concerns student feedback on building materials for a new university building in Herning.

*“We are aware that our emotions and knowledge regarding a subject may influence our viewpoints. A psychology unit from Aarhus University is interested in both your thoughts and feelings regarding these subjects. Therefore, as you provide your feedback, you will be asked about your thoughts and feelings several times during the survey. This also means that we expect emotional fluctuations, because one may react in different ways when expressing one’s opinion. It is important that you find a place where you can be alone and undisturbed. Please also make sure that you have a piece of paper and a pencil in front of you so that you can take notes during the survey.”*

***Anxiety first***

Induction of anxiety: “*Aarhus University has decided to actively join the debate about climate changes and approach the topic by taken the students’ opinions into account as it is important to the university that its actions reflect the opinions of its students. You are therefore invited to voice your opinion on this issue. In a little bit, you will be asked to give a short talk, stating your opinions on climate changes and how we may handle this issue at the university in the best way. We will walk you through the technical steps a bit later but essentially a red button will appear for you to click on after which, we will be recording your statements for 5 minutes. You will be asked to follow a link (https://online-voice-recorder.com), which you will be provided when you need it later on).*

*Members of the University Council will listen to all students’ taped arguments. The idea behind the sound recording is that a sound recording may be more convincing for the decision-makers than written arguments. Take a couple of minutes to write down a few statements as preparation for your speech to the experts.”*

Assessment 1:

Ratings of action tendencies, appraisals, and emotion experience outcomes. Paragraphs supporting the cover story are included: “*We realize that a task like this can have an emotional impact”. “Please rate how much knowledge you have on the field of climate changes on a scale from 1-5”. “On a scale from 1-5, do you consider yourself an expert on the topic?*”

Induction of anger:

*“Before considering the technical aspects of the presentation, we are also interested in getting your feedback on proposed changes in the economic student compensation system. A government-appointed committee has investigated ways in which universities in Denmark can oblige the government-demanded economic retrenchments. Instead of removing or reducing the student compensation system (as has been suggested several times before), the government-appointed committee has suggested that the universities should introduce a co-payment system. This implies that the students should pay a certain amount of money per obtained ECTS-points. A similar system already exists in more countries, including the US. Should the current proposal be implemented, this means that all students at Aarhus University must pay 500 DKK per ECTS-point, corresponding to 15.000 DKK per semester.*

*As expected, many student organizations have already expressed being upset about this proposed change. The organizations argue that such changes will create more inequality in our educational system. A number of meetings with the government-appointed committee have been planned in the weeks to come. In order to be as prepared as possible for these meetings, could you think of a few statements, expressing if and why the proposed changes are unfair – or perhaps even unjust. We will collect arguments from you and other university students.*

*Take a couple of minutes to write down a few personal notes in preparation. We will collect your feedback on the proposed changes in the economic student compensation system in the end of this survey. This collection will happen on Blackboard via a feature that anonymizes data.”*

Control condition:

*“Aarhus University has decided to build a new building in Herning for the engineers. It is important to the university that all such decisions take into account the aesthetics. There are two options for the building material, namely concrete and brick. The university would like to get your anonymous opinion on whether such a new building should be built in brick or concrete and why.*

*Take a couple of minutes to write down a few personal notes in preparation. We will collect your feedback on the new Buildings in Herning in the end of this survey. This collection will happen on Blackboard via a feature that anonymizes data.”*

Assessment 2: Ratings of action tendencies, appraisals, and emotion experience outcomes. Paragraphs supporting the cover story are included, for instance: ”*It is not possible to keep this survey open for a longer period of time, which is why we ask you to complete the rest of the survey before you are forwarded to the anonymous link*”.

Back to the anxiety condition (Assessment 3):

*“It is now time for you to give the talk on your opinions about the climate changes. Before doing so, please rate how you feel and what you feel like doing again. As you have just focused on something else, we would like to get information again as it may or may not have changed. Ratings of action tendencies, appraisals, and emotion experience outcomes. Paragraphs supporting the cover story are included.”*

***Debriefing***

*“Before giving the talk /writing your statements, the university would like to make sure that you understand the purpose of the study. In your own words, what is this study about? “*

After the participant’s response:

*“Now would be the time to give your talk and write your statements but this survey was actually a psychological experiment on how emotions can change the way in which we want to act in difficult situations. This means that there are* ***no*** *experts trying to eliminate the psychology students’ compensation system/building a new building in Herning, and there is no upcoming discussion concerning climate changes. Thus, you do not have to give a talk and you don’t have to provide statements. Please click on the box if you feel that you have been sufficiently informed about the nature of the study.”*

***Anger first***

In the Anger-first conditions, manipulations similar to the Anxiety-first conditions were used. Only the order of the manipulations differed:

- Overall introduction
- Induction of anger
- Assessment 1
- Induction of anxiety or control condition
- Assessment 2
- Back to the Anger condition (Assessment 3)
- Debriefing
